# Recent Progress in Dopant‐Free and Green Solvent‐Processable Organic Hole Transport Materials for Efficient and Stable Perovskite Solar Cells

**DOI:** 10.1002/advs.202307152

**Published:** 2024-02-28

**Authors:** Qinrong Cheng, Weijie Chen, Yaowen Li, Yongfang Li

**Affiliations:** ^1^ Laboratory of Advanced Optoelectronic Materials Suzhou Key Laboratory of Novel Semiconductor‐optoelectronics Materials and Devices College of Chemistry Chemical Engineering and Materials Science Soochow University Suzhou 215123 P. R. China; ^2^ Jiangsu Key Laboratory of Advanced Negative Carbon Technologies Soochow University Suzhou Jiangsu 215123 P. R. China; ^3^ State and Local Joint Engineering Laboratory for Novel Functional Polymeric Materials Jiangsu Key Laboratory of Advanced Functional Polymer Design and Application College of Chemistry Chemical Engineering and Materials Science Soochow University Suzhou 215123 P. R. China; ^4^ Beijing National Laboratory for Molecular Sciences CAS Key Laboratory of Organic Solids Institute of Chemistry Chinese Academy of Sciences Beijing 100190 P. R. China

**Keywords:** dopant‐free hole transport layer materials, perovskite solar cells, green solvents processable, stability

## Abstract

Dopant‐free hole transport layers (HTLs) are crucial in enhancing perovskite solar cells (pero‐SCs). Nevertheless, conventional processing of these HTL materials involves using toxic solvents, which gives rise to substantial environmental concerns and renders them unsuitable for large‐scale industrial production. Consequently, there is a pressing need to develop dopant‐free HTL materials processed using green solvents to facilitate the production of high‐performance pero‐SCs. Recently, several strategies have been developed to simultaneously improve the solubility of these materials and regulate molecular stacking for high hole mobility. In this review, a comprehensive overview of the methodologies utilized in developing dopant‐free HTL materials processed from green solvents is provided. First, the study provides a brief overview of fundamental information about green solvents and Hansen solubility parameters, which can serve as a guideline for the molecular design of optimal HTL materials. Second, the intrinsic relationships between molecular structure, solubility in green solvents, molecular stacking, and device performance are discussed. Finally, conclusions and perspectives are presented along with the rational design of highly efficient, stable, and green solvent‐processable dopant‐free HTL materials.

## Graphical abstract

Developing dopant‐free hole transport layer (HTL) materials processed with green solvents is essential for producing high‐performance perovskite solar cells. In this review, the intrinsic relationships between molecular structure, solubility, molecular stacking, and device performance are specifically discussed. It also proposes a molecular design guideline based on the Hansen solubility parameter principle to guide the green solvent‐processable dopant‐free organic HTL materials.

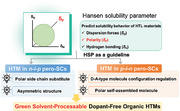

## 1 Introduction

Over the last decade, organometal halide perovskites have gained significant attention due to their unique photoelectric properties, such as high light absorption, a low exciton binding energy, and a long carrier‐diffusion length. The certified power conversion efficiencies (PCEs) of perovskite solar cells (pero‐SCs) have achieved an impressive 26.1%, thanks to the efforts of the global solar cell research community.^[^ ^1^, ^2^, ^3^, ^4^, ^5^, ^6^ ^]^ Typically, pero‐SCs feature a multilayer sandwiched device configuration, where charge transport layers are situated between the perovskite and electrodes, acting as buffer layers that enhance charge transport at the interface.^[^ ^7^, ^8^ ^]^ Hole transport layer (HTL) materials play pivotal roles in device performance, including hole extraction and transport, modification of the electronic band structure at the interface, and reduction of non‐radiative recombination loss.^[^ ^9^ ^]^ An ideal HTL material should also possess high hydrophobicity and photochemical stability to minimize perovskite degradation and ensure reliable long‐term operation of pero‐SCs.

Recently, a large number of HTL materials have been reported, including organic semi‐conductor materials^[^ ^10^, ^11^ ^]^ (small molecules and conducting polymer molecules) and inorganic ones^[^ ^12^ ^]^ (Ni‐based derivatives, Cu‐based derivatives, and transition metal oxides) based on their intrinsic nature. Although inorganic HTL materials feature the advantages of high stability, low cost, and high charge carrier mobility, a huge difficulty remains in preparing high‐quality HTLs. Most solvents for depositing inorganic HTL materials are intrinsically polar, which can partially dissolve and damage the perovskite film when fabricating conventional pero‐SCs. In addition, the necessary high‐temperature processing and the impurities in the inorganic HTL materials will deteriorate the performance and reproducibility of the devices, which limits the development and scope of inorganic HTL materials.^[^ ^13^, ^14^ ^]^ As a comparison, organic HTL materials with several notable advantages, such as fine‐tuned molecular structures, adjustable energy levels, low intrinsic defects, and easily tunable morphologies, have made substantial progress toward ideal HTLs.^[^ ^15^, ^16^ ^]^ Several state‐of‐the‐art organic HTL materials have been explored. For example, 2,2′,7,7′‐tetrakis [*N*,*N*‐di(4‐methoxyphenyl)amino]−9,9′‐spirobifluorene (Spiro‐OMeTAD) HTL has exhibited exceptional hole mobility and uniform film morphology when doped with lithium bis(trifluoromethane) sulfonimide (LiTFSI) and 4‐tert‐butylpyridine (*t*BP). This combination has consistently contributed to achieving record PCEs in pero‐SCs.^[^ ^17^, ^18^, ^19^, ^20^ ^]^ However, the hygroscopic LiTFSI and volatile *t*BP will inevitably degrade the perovskite layer and create pinholes in the HTL, thus reducing the device lifespan and yielding a suboptimal PCE. The more recently introduced *p*‐doped Poly[bis(4‐phenyl) (2,4,6‐trimethylphenyl) amine] (PTAA) stands out as an ideal HTL material for *p‐i‐n* devices due to its excellent film‐forming properties and minimal parasitic absorption. Although the amorphous morphology of PTAA is stable up to high temperatures, it is relatively vulnerable to allowing moisture ingress into the perovskite and somewhat prone to mechanical failure and delamination at interfaces. Commonly used F4TCNQ additive indeed enhance the device stability by hydrophobic fluorine atom incorporation and metal cation elimination, but the PCE is still unsatisfactory.^[^ ^21^, ^22^ ^]^ Furthermore, the additional doping processes add complexity and cost, making them unsuitable for large‐scale production of pero‐SCs. Hence, dopant‐free HTL materials are pivotal for improving the overall stability of pero‐SCs.

Dopant‐free HTL materials commonly suffer from intrinsic low hole mobility. Several strategies, such as introducing donor and acceptor units, expanding π‐conjugation, and enhancing the planarity, can promote tighter π‐π stacking for the molecules. This can effectively enhance the hole mobility of HTLs and, thus, device performance.^[^ ^23^, ^24^ ^]^ Until now, dopant‐free HTL‐based *n‐i‐p* pero‐SCs have achieved a record PCE of 24.53%, along with superior thermal and humidity stability thanks to the complete removal of unstable dopants.^[^ ^25^ ^]^ However, these dopant‐free HTL materials are usually processed using toxic aromatic solvents, such as chlorobenzene (CB) and chloroform (CF), which is problematic for large‐scale manufacturing. Using non‐toxic solvents such as ethyl acetate (EA), ethanol (EtOH), and acetone for processing leads to compromises in device performance primarily because of the limited solubility and crystallinity of HTL materials processed with these solvents.^[^ ^26^ ^]^ Solvent selection and rational molecule design are effective strategies to tackle this challenge.^[^ ^27^, ^28^ ^]^ In addition, the photoelectric and film properties of HTL materials in *p‐i‐n* pero‐SCs can be tuned easily by adjusting their molecular structure. For instance, the strong hydrophobicity of self‐assembled molecules that is unfavorable for perovskite deposition can be addressed in this way.^[^ ^29^, ^30^ ^]^ Recently, the Hansen solubility parameters (HSPs) were applied to organic semiconductor materials to determine and predict their solubility. They can guide the design of functional and green solvent‐processable HTL materials.^[^ ^31^ ^]^ In this way, some potential green solvents were selected, but only a few studies explored the relationship between the chemical structures and HSPs of these HTL materials.^[^ ^32^ ^]^ More studies in this area will facilitate the development of new HTL materials that are suitable for green processing.

**Figure 1:**
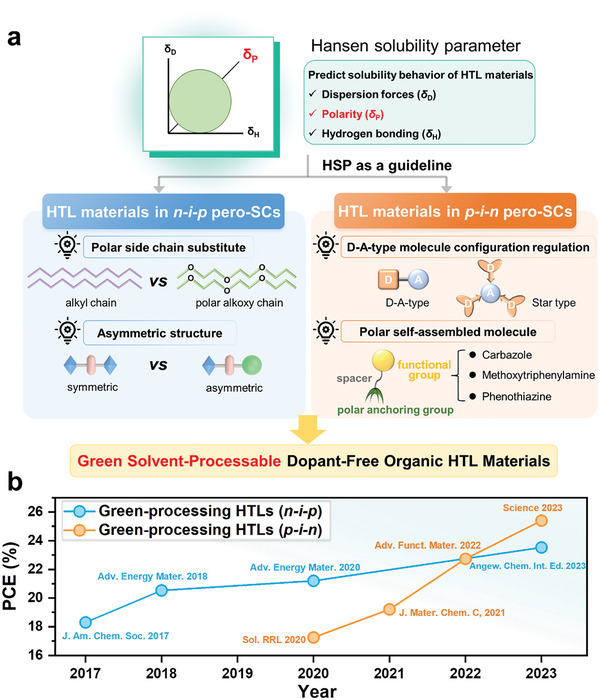
a) HSP principle as a molecular design guideline for green solvent‐processable dopant‐free organic HTL materials. b) PCE evolution based on green solvent‐processable HTL materials utilized in pero‐SCs (some representative studies).

In this review, we have summarized the currently available green solvent‐processable dopant‐free HTL materials that are suitable for building high‐performance pero‐SCs. It also proposes a molecular design guideline based on the HSP principle to guide the green solvent‐processable dopant‐free organic HTL materials (**Figure** **1**a). The PCE evolution (Figure 1b) indicates the skyrocketing improvement of pero‐SCs based on green solvent‐processable HTL materials. This review primarily encompasses two key sections: solvent selection and molecular structure design (Figure 1a). In the solvent selection section, we briefly discuss basic considerations in green solvent‐processable dopant‐free HTL materials, including the determination of HSP, properties of processing solvents according to HSP theory, and key challenges. In the molecular structure design section, we thoroughly summarize and discuss the development of green solvent‐processable dopant‐free HTL materials for different device structures. More specifically, we focus on the design principles of these materials, including polar side chain substitution, asymmetric structure, donor–acceptor (D‐A) type configuration regulation, and polar self‐assembled molecules. Finally, we provide a summary of the highlights of this work and perspective on future research toward realizing highly efficient, stable, and green solvent‐processable HTL materials.

## 2 Fundamental Information About Green Solvents

### 2.1 Classification of Solvents

As is widely known, there are a large number of organic solvents, and each solvent's toxicity depends on its specific chemical structure. Green solvents generally have very low toxicity and should not be harmful to humans and the environment. It is important to clarify that the term “green solvent,” as discussed here, relates to a comparison with traditional toxic halogenated solvents rather than aligning with the well‐defined green solvents of green chemistry.^[^ ^33^ ^]^ In general, low‐toxicity solvents, such as chlorine‐free solvents, non‐halogenated and non‐aromatic solvents, and alcohols, are promising alternatives to the current widely used hazardous solvents.

**Figure 2:**
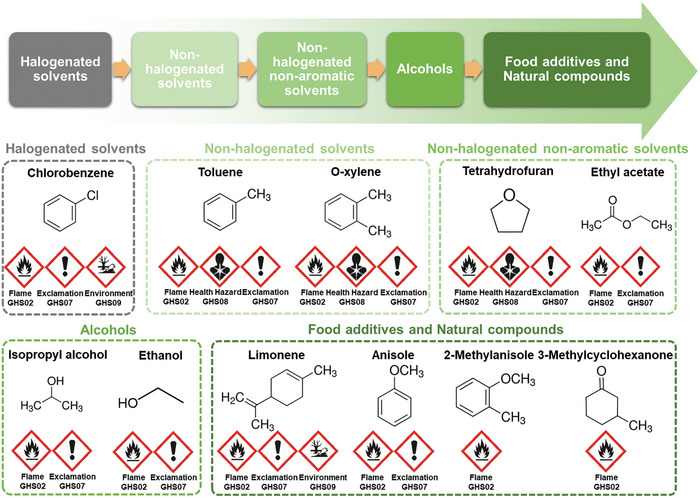
Green solvent classes (top arrow) and MSDS hazard symbols for various solvents.

The accumulation of halogen atoms can be hazardous to humans and ecosystems. Therefore, non‐halogenated solvents should be considered first as green solvent candidates. Nonetheless, some non‐halogenated (e.g., toluene (TL) and *o*‐xylene) and non‐aromatic solvents (e.g., tetrahydrofuran (THF) and EA) may still carry potential toxicity concerns.^[^ ^34^ ^]^ More recently, water, alcohols, food additives, and natural compounds have been used as non‐toxic green solvents for organic solar cells (OSCs).^[^ ^35^, ^36^ ^]^ Water is commonly considered to be the best green solvent because it is not toxic and low‐cost. However, water is typically not an ideal solvent for the preparation of pero‐SCs because it can damage the lattice structure of perovskite.^[^ ^37^ ^]^ Fortunately, some alcohols (e.g., isopropyl alcohol (IPA) and EtOH), food additives (e.g., anisole (ANS) and 2‐methylanisole (2‐MA)), and natural compounds (e.g., 3‐methylcyclohexanone (3‐MC)) are virtually harmless to humans or the environment according to their material safety data sheets (MSDSs) – see **Figure** **2**. This is because they do not carry the symbols GHS05 (corrosive), GHS06 (toxic), GHS08 (health hazard), or GHS09 (environmental hazard). Therefore, these solvents are suitable for mass production even if they are classed as GHS02 (flammable).^[^ ^38^ ^]^ This review summarizes the dopant‐free HTL materials, which can be processed from low‐toxic solvents and non‐toxic green solvents and can serve as a reference for further advances of green solvent‐processable dopant‐free HTL materials.

### 2.2 Properties of Solvents According to HSP Theory

To obtain a high‐performance green solvent‐processable HTL, it is crucial to ensure that these materials dissolve well in green solvents, which facilitates the formation of a high‐quality film. HSP help describe and predict the solubility of organic semiconductors in specific solvents. It comprises three aspects: dispersive interactions, polar interactions, and hydrogen‐bonding interactions. The dispersive forces consist of interactions between induced dipoles (London forces), while forces between permanent dipoles (Keesom forces) and forces between permanent and induced dipoles (Debye forces) are included in the polar forces. The following equation can be used:

(1) $$\delta \;={\left(\delta_{D}^{2}{+\delta }_{P}^{2}{+\delta }_{H}^{2}\right)}^{1/2}$$

**Figure 3:**
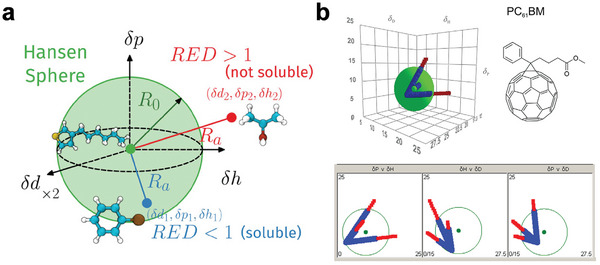
a) Schematic representation of the Hansen space. Reproduced with permission.^[^ ^39^ ^]^ Copyright 2018, Wiley‐VCH. b) 3D representation of the HSPs model of PC_61_BM. Reproduced with permission.^[^ ^40^ ^]^ Copyright 2021, Wiley‐VCH.

Here, *δ*_D_, *δ*_P_, and *δ*_H_ denote the dispersion, polar, and hydrogen‐bonding components, respectively.^[^ ^41^ ^]^ The HSP coordinates of the solute are determined by analyzing its solubility in a series of solvents with known HSPs (**Figure** **3**a). A fitted spheroid within the solubility space can be visualized using a three‐dimensional coordinate system (Hansen space) with the axes *δ*_D_, *δ*_P_, and *δ*_H_ (Figure 3b). Following this procedure, the center of the sphere denotes the three Hansen parameters of the solute, while the radius of the sphere (*R*_0_) represents the interaction radius for the solute, where good solvents lie within the spheroid and non‐solvents outside.

Another important parameter, *R*_a_, is defined as the radius of interaction. It can be determined using the HSPs difference between solvent and solute. *R*_a_ can be calculated using Equation (2) with a perfector of 4 for the dispersive interaction term:

(2) $$R_{a}^{2}=\;4{\left(\delta_{D1}-\delta_{D2}\right)}^{2}+{\left(\delta_{P1}-\delta_{P2}\right)}^{2}+{\left(\delta_{H1}-\delta_{H2}\right)}^{2}$$

Here, the numbers 1 and 2 in the subscripts represent the solute and solvent, respectively. A small *R*_a_ value indicates there are similar chemical characteristics between solute and solvent, which means the solute is likely to dissolve in the solvent. If *R*_a_ is divided by *R*_0_, a useful parameter, known as relative energy difference (RED), can be obtained (*eq* 3). When RED < 1, there is a high chance for the solute to dissolve in the solvent, while they are unlikely to mix when RED > 1.

(3) $$\mathrm{RED}\;=\frac{R_{a}}{R_{0}}$$

Typically, halogenated aromatic solvents, such as CB, show high *δ*_D_, low *δ*_P_, and low *δ*_H_ values (**Table** **1**). In contrast, ideal green solvents (e.g., alcohol, food additives, and natural compounds) typically have higher *δ*_P_ and *δ*_H_ values and a similar *δ*_D_ compared to halogenated solvents. To enhance the solubility of dopant‐free HTL materials in green solvents, it is necessary to precisely adjust their molecular structures to align with the HSPs of these green solvents. This follows the fundamental principle of “like dissolves like”.^[^ ^42^ ^]^

**Table 1:** Hansen parameters of different solvents.

Solvents	*δ*_D_ (MPa^0.5^)	*δ*_P_ (MPa^0.5^)	*δ*_H_ (MPa^0.5^)
Halogenated solvents			
Chlorobenzene (CB)	19	4.3	2
Non‐halogenated solvents			
Toluene (TL)	18	1.4	2
O‐xylene	17.8	1	3.1
Non‐halogenated non‐aromatic solvents			
Tetrahydrofuran (THF)	16.8	5.7	8
Ethyl acetate (EA)	15.8	5.3	7.2
Alcohols			
Isopropyl alcohol (IPA)	15.8	6.1	16.4
Ethanol (EtOH)	15.8	8.8	19.4
Food additives and Natural compounds			
Limonene	17.2	1.8	4.3
Anisole (ANS)	17.8	4.4	6.9
2‐Methylanisole (2‐MA)	18.3	4.7	4.8
3‐Methylcyclohexanone (3‐MC)	17.7	7.7	4.7

### 2.3 Key Challenges in Green Solvent‐Processable Dopant‐Free HTL Materials

To obtain novel green solvent‐processable dopant‐free HTL materials for efficient and stable pero‐SCs, several major challenges need to be overcome. Typically, HTL materials should have a large π‐conjugated framework and suitable molecular dipoles to ensure strong intermolecular interactions that enable high hole mobilities in *n‐i‐p* pero‐SCs. Unfortunately, this weakens the interaction between HTL materials and solvent molecules, which reduces the solubility and film‐forming properties in green solvents, such as ANS, EtOH, 2‐MA, and EA.^[^ ^43^, ^44^ ^]^ Thus, a significant trade‐off between achieving high hole mobility and enabling green solvent processing has persisted within this field for an extended period. It is crucial to solve the tradeoff issue between the high crystallinity of HTL materials and high solubility processed with green solvents through reasonable molecular design. In *p‐i‐n* pero‐SCs, the above trade‐off challenge still remains to be solved for D‐A‐type HTL materials. Another challenge is that the self‐assembled molecules commonly exhibit hydrophobic properties, which lowers the wettability of perovskite precursor solution. Typically, a substrate with good wettability is particularly important to produce high‐quality and large‐scale perovskite films.^[^ ^45^, ^46^ ^]^

## 3 Green Solvent‐Processable Dopant‐Free HTL Materials for *n*‐*i*‐*p* Pero‐SCs

**Figure 4:**
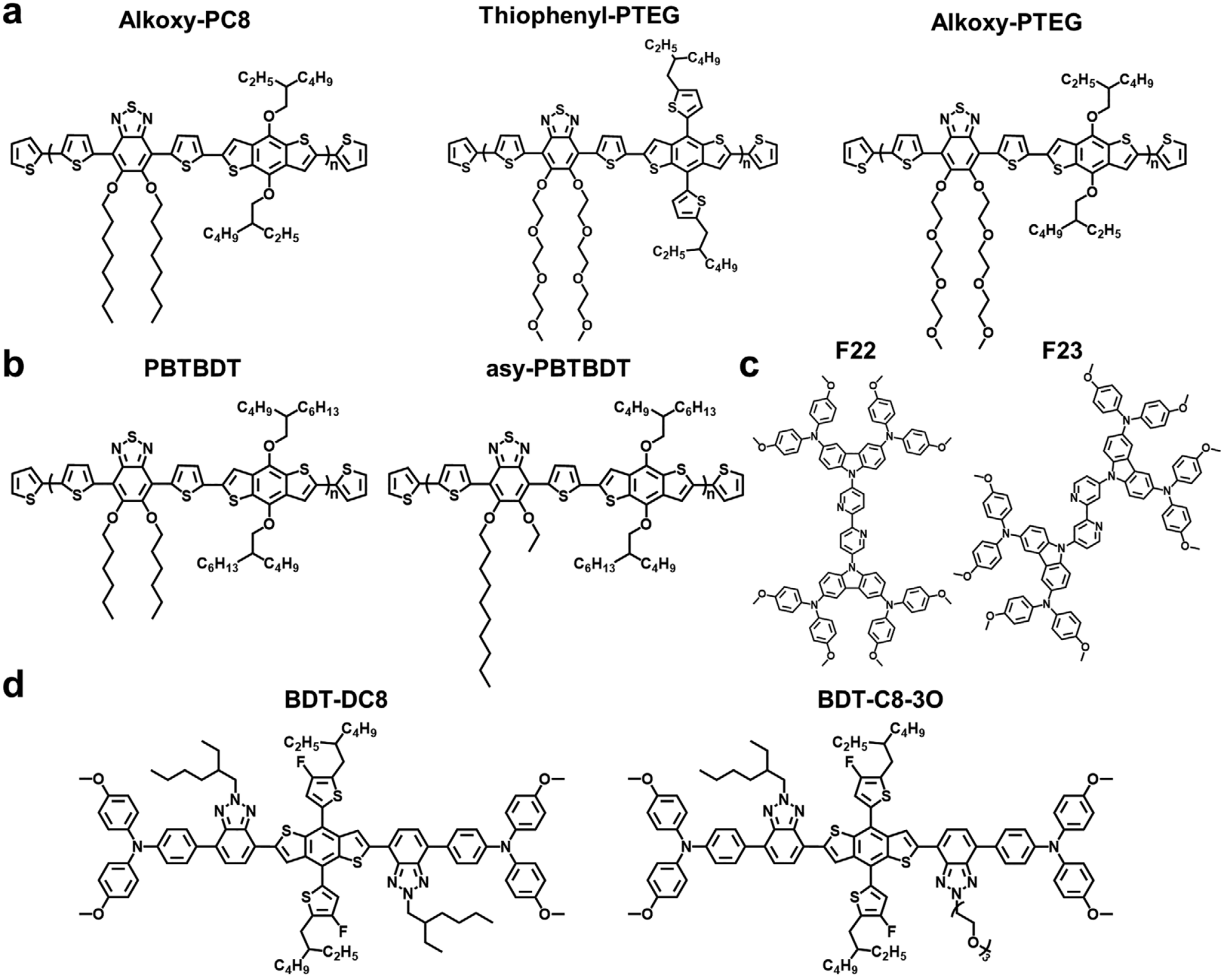
Molecular structures of green solvent‐processable dopant‐free HTL materials in *n*‐*i*‐*p* pero‐SCs.

To date, the design philosophy for creating dopant‐free HTL materials that are compatible with green solvents remains somewhat unclear. It was reported that increasing the length or number of alkyl substituents can improve the solubility of HTL materials in green solvents. However, the large steric hindrance of alkyl chains will hinder the intermolecular interactions between the conjugated main chain, thus decreasing the hole mobility.^[^ ^47^, ^48^ ^]^ To address this problem, recent approaches can be divided into two categories: 1) appending functional side chains instead of the conventional alkyl chains to the conjugated main chains,^[^ ^49^, ^50^ ^]^ such as polar side chain, and 2) introducing an asymmetric structure. These two methods can weaken the intermolecular interaction between polymer chains and effectively increase the solubility of HTL materials. **Figure** **4** and **Table** **2** summarize the molecular structures of reported green solvent‐processable HTL materials for *n‐i‐p* pero‐SCs and associated photovoltaic parameters.

**Table 2:** Detailed photovoltaic parameters of green solvent‐processable HTL materials utilized in *n*‐*i*‐*p* pero‐SCs.

HTL material	Solvent	*V*_oc_ [V]	*J*_sc_ [mA cm^−2^]	FF [%]	PCE [%]	Ref.
alkoxy‐PTEG	3‐MC	1.14	23.2	79.8	21.2	[51]
asy‐PBTBDT	2‐MA	1.11	22.4	73.2	18.3	[52]
F23	THF	1.07	21.6	76.1	17.6	[54]
BDT‐C8‐3O	3‐MC	1.160	25.4	79.87	23.53	[55]
Spiro‐OMeTAD	THF	1.02	21.3	77.8	16.94	[58]
Spiro‐OMeTAD	*p*‐xylene	1.11	23.3	72.0	18.66	[59]
Spiro‐OMeTAD	EA	1.12	22.9	75.6	19.43	[60]
Spiro‐OMeTAD	ANS	1.14	23.9	76.0	20.53	[61]

### 3.1 Polar Side Chain Substitute

Since green solvents commonly feature a high *δ*_P_, the HTL molecules that are compatible with green solvents should also have a high *δ*_P,_ considering the “like‐dissolves‐like” principle. Moreover, polar substituents can effectively increase the *δ*_P_ of molecules, which can enhance the solubility of these materials in green solvents by overcoming the strong *π–π* stacking of the molecular backbone.

**Figure 5:**
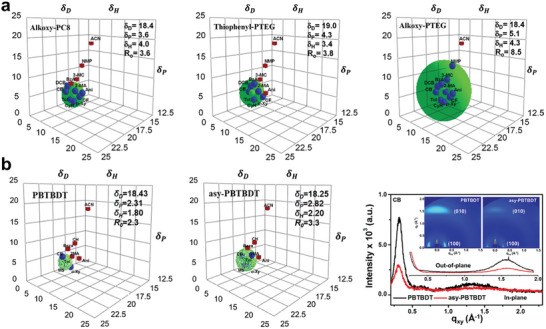
a) HSPs of alkoxy‐PC8, thiophenyl‐PTEG, and alkoxy‐PTEG. Reproduced with permission.^[^ ^51^ ^]^ Copyright 2020, Wiley‐VCH. b) HSPs and GIWAXS of PBTBDT and asy‐PBTBDT. Reproduced with permission.^[^ ^52^ ^]^ Copyright 2017, American Chemical Society.

For example, Park et al. reported a series of novel D–A type polymers with different side chains (Figure 4a).^[^ ^51^ ^]^ The substitution of thiophene groups with alkoxy groups can improve the solubility of alkoxy‐PTEG in non‐aromatic green solvents because the hydrophilic alkoxy groups can increase the polarity. Accordingly, the calculated HSP parameters of alkoxy‐PTEG (*δ*_D_ = 18.4, *δ*_P_ = 5.1, *δ*_H_ = 4.3 MPa^0.5^) are closer to those of the green solvents (2‐MA: *δ*_D_ = 18.3, *δ*_P_ = 4.7, *δ*_H_ = 4.8 MPa^0.5^ and 3‐MC: *δ*_D_ = 17.7, *δ*_P_ = 7.7, *δ*_H_ = 4.7 MPa^0.5^) than alkoxy‐PC8 (*δ*_D_ = 18.4, *δ*_P_ = 3.6, *δ*_H_ = 4.0 MPa^0.5^) and thiophenyl‐PTEG (*δ*_D_ = 19.0, *δ*_P_ = 4.3, *δ*_H_ = 3.4 MPa^0.5^) (**Figure** **5**a). Furthermore, alkoxy‐PTEG shows superior solubility (*R*_0_ = 8.5) compared to alkoxy‐PC8 (*R*_0_ = 3.6) and thiophenyl‐PTEG (*R*_0_ = 3.8). As such, alkoxy‐PTEG exhibited superior solubility in 2‐MA and 3‐MC, indicating sufficient solubility in each solvent (>15 mg mL^−1^). The resulting alkoxy‐PTEG‐based pero‐SC exhibited PCEs of 21.2% and 19.9% when processed from 2‐MA and 3‐MC, respectively. In addition, the pero‐SC retained 88% after 30 days at ambient conditions (40‐50% relative humidity and room temperature).

### 3.2 Asymmetric Structures

Because low crystallinity can result in high solubility in green solvents,^[^ ^53^ ^]^ the introduction of asymmetric structure is a novel approach to increase solubility by suppressing molecule aggregation and crystallinity. Compared with previous methods, such as bulky or polar side chain substitution, the introduction of an asymmetric side chain can sustain the high hole mobility of the molecules by maintaining the extended conjugation of the backbone. In other words, asymmetric side chains can maintain the high crystallinity of molecules while improving their solubility in green solvents.

In 2017, Park et al. reported two polymeric HTL materials, D‐A‐type PBTBDT and asy‐PBTBDT, which mainly consist of benzothiadiazole (BT) and benzo[1,2‐ b:4,5:b′]dithiophene (BDT) units (Figure 4b).^[^ ^52^ ^]^ With respect to the side chain on the BT unit, an asymmetric alkyl substituent was adopted to substitute conventional symmetric alkyl side chains for obtaining asymmetric molecule asy‐PBTBDT. The HSP results indicate that asy‐PBTBDT (*δ*_D_ = 18.25, *δ*_P_ = 2.82, *δ*_H_ = 2.20 MPa^0.5^, and *R*_0_ = 3.3) has a higher polarity and solubility capacity than symmetric PBTBDT (*δ*_D_ = 18.43, *δ*_P_ = 2.31, *δ*_H_ = 1.80 MPa^0.5^, and *R*_0_ = 2.3) (Figure 5b). According to grazing‐incidence wide‐angle X‐ray scattering (GIWAXS) results, the crystallinity of asy‐PBTBDT films is lower than that of PBTBDT. It can be attributed to the presence of asymmetric alkyl substituents on the BT unit, which introduces irregularities into the asy‐PBTBDT repeating units and effectively alleviates the excessive aggregation. As a result, asy‐PBTBDT has excellent solubility even in 2‐MA. Because it still maintains the conjugated backbone length, asy‐PBTBDT still maintains high crystallinity and achieves high hole mobility even without the use of dopants. The pero‐SCs based on 2‐MA‐processed asy‐PBTBDT HTL exhibited a PCE of 18.3%. The unencapsulated device retained 91% of the initial PCE after 30 days at ambient conditions under 50−75% relative humidity. Compared to asymmetric conjugated polymers, asymmetric small molecules possess low molecular weight, a definite chemical structure, larger dipole moment, and stronger binding energy. These properties make them more susceptible to being dissolved in a wider variety of green solvents. For example, Zhu et al. synthesized two new asymmetric small molecule HTL materials, in F23, the carbazole‐diphenylamine was modified on the 4,4′‐position of the 2, 2′‐bipyridine group, while the carbazole‐diphenylamine group was substituted on the 5, 5′‐position of the bipyridine core structure in F22 (Figure 4c).^[^ ^54^ ^]^ The bipyridine, with its strong electron‐withdrawing properties, can enhance the intramolecular charge transfer from carbazole‐diphenylamine to bipyridine. This increase in intramolecular charge transfer can enhance both molecular polarity and intermolecular interactions and thus promote intermolecular charge transfer. The carbazole‐diphenylamine group grafted on different positions of bipyridine moiety make F22 and F23 show different molecular configurations, charge delocalization, and energy levels. F23 exhibits a smaller reorganization energy and higher hole mobility than those of F22. As a result, the (non‐halogenated solvent) THF‐processed dopant‐free F23 film showed a uniform morphology, which effectively reduced the charge recombination and enhanced interfacial charge transfer. The resulting device exhibited a PCE of 17.60% and enhanced stability. In contrast, the device based on undoped F22 shows a much inferior PCE of 15.31%.

The incorporation of a polar asymmetric side chain features the advantages of the above two strategies, which is promising to improve the solubility of HTL materials in the green solvent while maintaining their high crystallinity. Li et al. designed a novel, linear, organic small molecule BDT‐C8‐3O by introducing an asymmetric polar oligo(ethylene glycol) (OEG) side chain (Figure 5c).^[^ ^55^ ^]^ This method not only overcomes the solubility limitations in green solvents but also enables stacking the conjugated main chains in two patterns, which further enhances crystallinity and hole mobility. As a result, the *n‐i‐p* pero‐SCs based on 3‐MC‐processed BDT‐C8‐3O HTL that without any dopant delivered outstanding PCEs of 23.53%.

### 3.3 Green Solvent‐Processable Dopant‐Free Spiro‐OMeTAD

As a state‐of‐the‐art HTL material, Spiro‐OMeTAD has always dominated the PCE of typical *n*‐*i*‐*p* pero‐SCs. It is well known that Spiro‐OMeTAD is commonly processed from a highly toxic solvent, CB, which is undesirable for industrial manufacturing.^[^ ^56^, ^57^ ^]^ Therefore, environmentally friendly solvents are urgently needed to process Spiro‐OMeTAD. Recently, Yan et al. first reported a 16.94%‐efficiency‐pero‐SCs that used THF‐processed dopant‐free Spiro‐OMeTAD,^[^ ^58^ ^]^ which also showed a negligible hysteresis effect. The THF‐processed Spiro‐OMeTAD film showed enhanced crystallinity and higher hole mobility than the CB‐processed one. In addition, only 5 mg mL^−1^ Spiro‐OMeTAD in THF was required to cover the perovskite layer, which is much lower than the concentration needed with CB (70 mg mL^−1^). Later, Fu et al. increased the PCE to 18.66% by blade coating the Spiro‐OMeTAD HTL using low toxic solvents (*p*‐xylene) in ambient air.^[^ ^59^ ^]^

**Figure 6:**
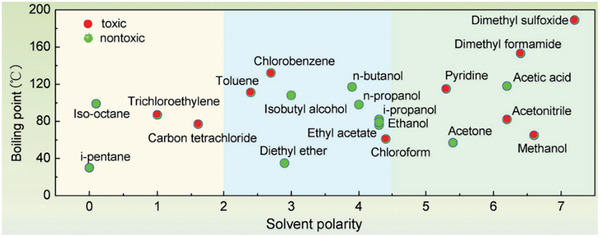
Statistical diagram showing polarities and boiling points of commonly used solvents. Reproduced with permission.^[^ ^60^ ^]^ Copyright 2017, Wiley‐VCH.

To systematically choose green solvents for Spiro‐OMeTAD, it becomes imperative to develop a model capable of rapid and precise screening. Zhong et al. screened many industrial solvents to find suitable green solvent candidates.^[^ ^60^ ^]^ Following extensive experimental investigations, it was discovered that solvents falling within the polarity range of 2.0 to 4.5 are potential candidates for green solvents (as shown in **Figure** **6**). The polarity here refers to the polarity of the solvent molecule, that is, the uneven degree of positive and negative charge distribution within the molecule. The greater the polarity, the higher the degree of uneven distribution of positive and negative charges inside the solvent molecule. Notably, solvents such as CB, TL, CF, acetonitrile (ACN), pyridine (PY), dimethyl formamide (DMF), dimethyl sulfoxide (DMSO), gamma‐butyrolactone (GBL), and N‐methyl pyrrolidone (NMP) with polarities exceeding 2.0 exhibited the capacity to dissolve or partially dissolve Spiro‐OMeTAD. Considering the Spiro‐OMeTAD HTL is typically coated on the perovskite film, DMF, DMSO, GBL, and NMP with high polarities > 4.5 are not suitable solvents because they would dissolve the perovskite. In addition, ACN and PY can also decompose the perovskite. Therefore, the green solvent, EA, which has a polarity of 4.3, was selected to replace CB to dissolve Spiro‐OMeTAD. EA‐processed Spiro‐OMeTAD films have less solvent residue due to the lower boiling temperature (77 °C) of EA. As a result, few pinholes were observed in the EA‐processed Spiro‐OMeTAD films, which greatly reduce the possible charge recombination, thus exhibiting faster carrier transport and lower defects than CB‐processed devices. These advances increased the PCE of the EA‐processed device to 19.43%, which is significantly higher than for the CB‐processed device (PCE = 18.67%). More recently, Saliba et al. proposed ANS as a more environmentally friendly solvent that is used as a precursor for perfume manufacturing to dissolve Spiro‐OMeTAD (90 × 10^−3^ m).^[^ ^61^ ^]^ The relevant pero‐SCs showed a remarkable PCE of 20.53%, which is comparable to that of the CB‐processed devices.

Based on the above works, we summarized and proposed green solvent‐processable molecular design guidelines following the principle of “like dissolves like”. Two corresponding modification strategies were summarized to realize the asymmetric transformation for HTL materials, which can increase the molecule polarity without changing its conjugation: 1) The asymmetric modification of the conjugated central backbone. Regarding the backbone modification, changing the sequence, species, or length of the central fused ring in HTL materials can lead to variations in their molecular packing, energy levels, and dipole moments, while maintaining relative π‐electron delocalization. This approach has shown great potential in improving device performance. 2) The introduction of polar side chains. The side chains play a crucial role in achieving a balance between solubility, charge transport, and crystallization behavior. Different from symmetrical modification of the side chain structure, one‐sided modification might be more conducive to making a subtle adjustment of the properties mentioned above, such as the introduction of OEG, amino, phosphonate side chains, etc.^[^ ^28^ ^]^ Increasing the polarity of HTL materials by incorporating polar substituents can increase the *δ*_P_ value and enhance their solubility capacity in green solvents. These strategies can effectively promote the development of green solvent‐processable HTL materials that possess desired properties, thereby providing greater accuracy in supporting materials selection.

## 4 Green Solvent‐Processable Dopant‐Free HTL Materials for *p*‐*i*‐*n* Pero‐SCs

In *p*‐*i*‐*n* pero‐SCs, the terminated HTL material on the substrate can act as a template for the subsequent deposition and crystallization of perovskite. These HTL materials located on the light‐incident side should be as broadband transparent as possible with regard to photo transmission to avoid current loss in the device. In addition, green solvent‐processable dopant‐free HTL materials remain highly desirable to obtain high‐performance *p*‐*i*‐*n* pero‐SCs and enable large‐scale production. Several approaches have been proposed to enable the use of green solvent‐processable HTL materials, including D–A‐type small molecule configuration regulation and polar self‐assembled molecules.

### 4.1 D–A Type Molecule Configuration Regulation

**Figure 7:**
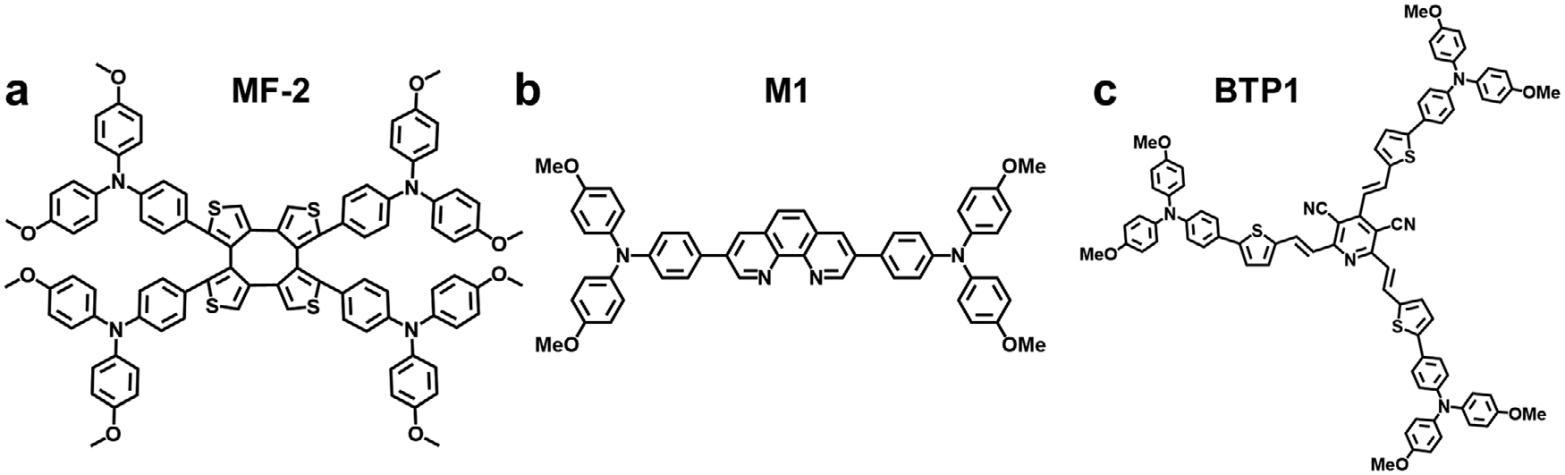
Molecular structures of green solvent‐processable D–A‐type small molecule HTL materials in *p*‐*i*‐*n* pero‐SCs.

Utilizing a D‐A structured backbone and expanding the π conjugation within small molecules are effective strategies for improving the charge extraction properties of the HTL. These approaches take advantage of the inherent polarity and high dipole moment of these molecules, which can induce self‐doping and a built‐in potential, respectively, which facilitates charge extraction.^[^ ^10^, ^62^ ^]^ **Figure** **7** and **Table** **3** provide an overview of the molecular structures of green solvent‐processable D‐A‐type small molecule HTL materials for *p‐i‐n* pero‐SCs and detailed photovoltaic parameters, respectively.

**Table 3:** Detailed photovoltaic parameters of green solvent processable D‐A‐type small molecule HTL materials utilized in *p*‐*i*‐*n* pero‐SCs.

HTL material	Solvent	*V*_oc_ (V)	*J*_sc_ (mA cm^−2^)	FF (%)	PCE (%)	Ref.
MF‐2	EA	1.04	21.4	77.7	17.25	[63]
M1	EA	1.07	21.8	82.5	19.21	[64]
BTP1	2‐MA	1.18	24.95	82.83	24.34	[65]

**Figure 8:**
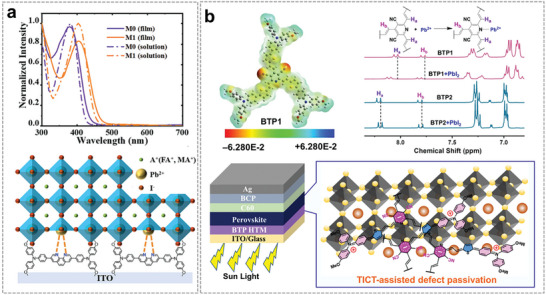
a) UV–vis absorption spectrum of M1 in solution and thin films, and schematic diagram of the M1/perovskite interfaces. Reproduced with permission.^[^ ^64^ ^]^ Copyright 2021, Royal Society of Chemistry. b) Calculated ESP map based on the density functional theory (DFT) method and Schematic diagram of BTP1 passivating defects at the buried interface (“twisted intermolecular charge transfer” is defined as “TICT”). Reproduced with permission.^[^ ^65^ ^]^ Copyright 2023, Wiley‐VCH.

Recently, Li et al. developed a new HTL material, MF‐2, which consists of a cyclooctatetrathiophene (COTh) core and four methoxytriphenylamine (MPA) arms (Figure 7a).^[^ ^63^ ^]^ Rotating single bonds within the COTh core can optimize the orientation of thiophene units, thereby enhancing the π‐π stacking of the MPA arms. Notably, MF‐2 exhibits good solubility in the green solvent EA because the S in its flexible core COTh can interact with O in EA. The resulting devices, which were based on EA‐processed MF‐2, exhibited a PCE of 17.25%. Guo et al. developed a new HTL material, M1, that is both cost‐effective and green solvent‐processable. This molecule involved engaging a planar bidentate ligand with highly polar moieties as the core structure (Figure 7b).^[^ ^64^ ^]^ The red‐shifted absorption spectra of the solid film show that such an HTL can facilitate π‐π stacking (**Figure** **8**a). In addition, the nitrogen atoms on the 1,10‐phenanthroline in M1 can effectively interact with the Pb^2+^ defects at the M1/perovskite interface, which significantly reduces non‐radiative recombination. The obtained device based on CB‐processed dopant‐free M1 exhibited a PCE of 20.14%. More importantly, the device, which was made with the green solvent EA‐processed M1, could also maintain a high PCE of 19.21%. In addition, a higher storage stability of the M1‐based devices was achieved.

The above D–A‐type molecule will inevitably sacrifice the solubility and film‐forming ability in green solvents thanks to the strong dipole–dipole interactions between the molecules. Integrating the D–A conjugation with a star‐shaped structure could be a viable solution to address the trade‐off between high crystallinity and solubility due to its bulky nonplanar structure. However, achieving success in this regard has proven challenging, as the majority of star‐shaped D–A‐type HTL materials are constructed using A‐D‐A‐type structures.^[^ ^66^ ^]^ The highly planar ring structure of the A units can easily lead to strong terminal‐terminal and/or arm–arm interactions, which leads to excessive aggregation.^[^ ^67^, ^68^ ^]^ To mitigate undesired molecular aggregates, peripheral D units with twisted configurations, such as triphenylamine (TPA), were introduced. Given that the D‐A‐D structure is more conducive to hole transport,^[^ ^69^, ^70^, ^71^ ^]^ it is preferred to construct star‐shaped D‐A‐D type HTL materials with the A unit as the core.

Recently, Li et al. have successfully demonstrated that star‐shaped D–A–D structural design is an effective approach toward efficient green solvent processable dopant‐free HTL materials (Figure 7c).^[^ ^65^ ^]^ The developed BTP‐1 molecule, with the electron‐deficient 3,5‐dicarbonitrile pyridine (DCP) unit as the core, exhibits comparable HSP values (*δ*_D_ = 18.49, *δ*_P_ = 12.93, *δ*_H_ = 3.52 MPa^0.5^, *R*_0_ = 13.7) as for 2‐MA, which suggests good solubility in 2‐MA. Furthermore, the obtained dopant‐free BTP1 HTL showed good surface wettability, suitable energy level, and high hole mobility, which facilitated the growth of perovskites as well as rapid interfacial charge extraction. In addition, BTP1 can effectively passivate defects at the buried interface due to the strong interaction between BTP1 and uncoordinated Pb^2+^ (Figure 8b). The resulting *p*‐*i*‐*n* pero‐SCs based on 2‐MA processed‐BTP1 HTL featured a high PCE of 24.34%. This represents the highest value for green solvent processable D–A‐type small molecule HTL‐based devices. The unencapsulated devices of BTP1 can further remain over 98% of initial PCE after storing in an N_2_ atmosphere under 1 sun illumination for 1000 h.

Above all, green solvent‐processable molecular design guideline based on the HSPs principle was summarized. To mitigate undesired molecular aggregates, integrating the D–A conjugation with a star‐shaped structure can address the trade‐off issue between high crystallinity and solubility due to its bulky nonplanar structure. This strategy can improve the solubility and film‐forming ability of HTL materials in green solvents. To maintain a high hole mobility, peripheral D units with twisted configurations, such as TPA or diphenylamine can also be introduced.^[^ ^15^ ^]^ In addition, by regulating the central unit or developing new central units, researchers can construct multi‐arm three‐dimensional configuration molecules with three‐dimensional conjugation and charge transport. For example, the incorporation of side chain and end group fluorination can introduce fluorine atoms in both horizontal and vertical directions of the molecule skeleton. By inducing multiple weak intermolecular interactions, a three‐dimensional network with close packing can be constructed, which facilitates efficient three‐dimensional exciton and charge transport.^[^ ^72^ ^]^

### 4.2 Polar Self‐Assembled Molecule

In the scope of future high‐throughput commercialization, the facile synthesis and deposition of HTL materials with low cost are highly desired for large‐area device fabrication. These requirements can be easily met for self‐assembled monolayers (SAMs) due to their simple structures and unique self‐assembly properties.^[^ ^73^ ^]^ SAMs are currently regarded as novel HTL material candidates for *p*‐*i*‐*n* type pero‐SCs featuring both high efficiency and high stability.

**Figure 9:**
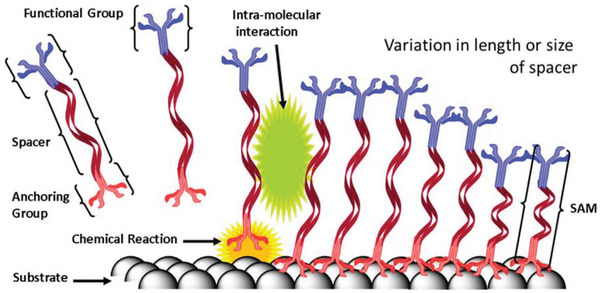
Three parts in SAM and the schematic diagram for the mechanism of SAMs showing the chemical interaction between an anchoring group and substrate. Reproduced with permission.^[^ ^74^ ^]^ Copyright 2020, Wiley‐VCH.

**Figure 10:**
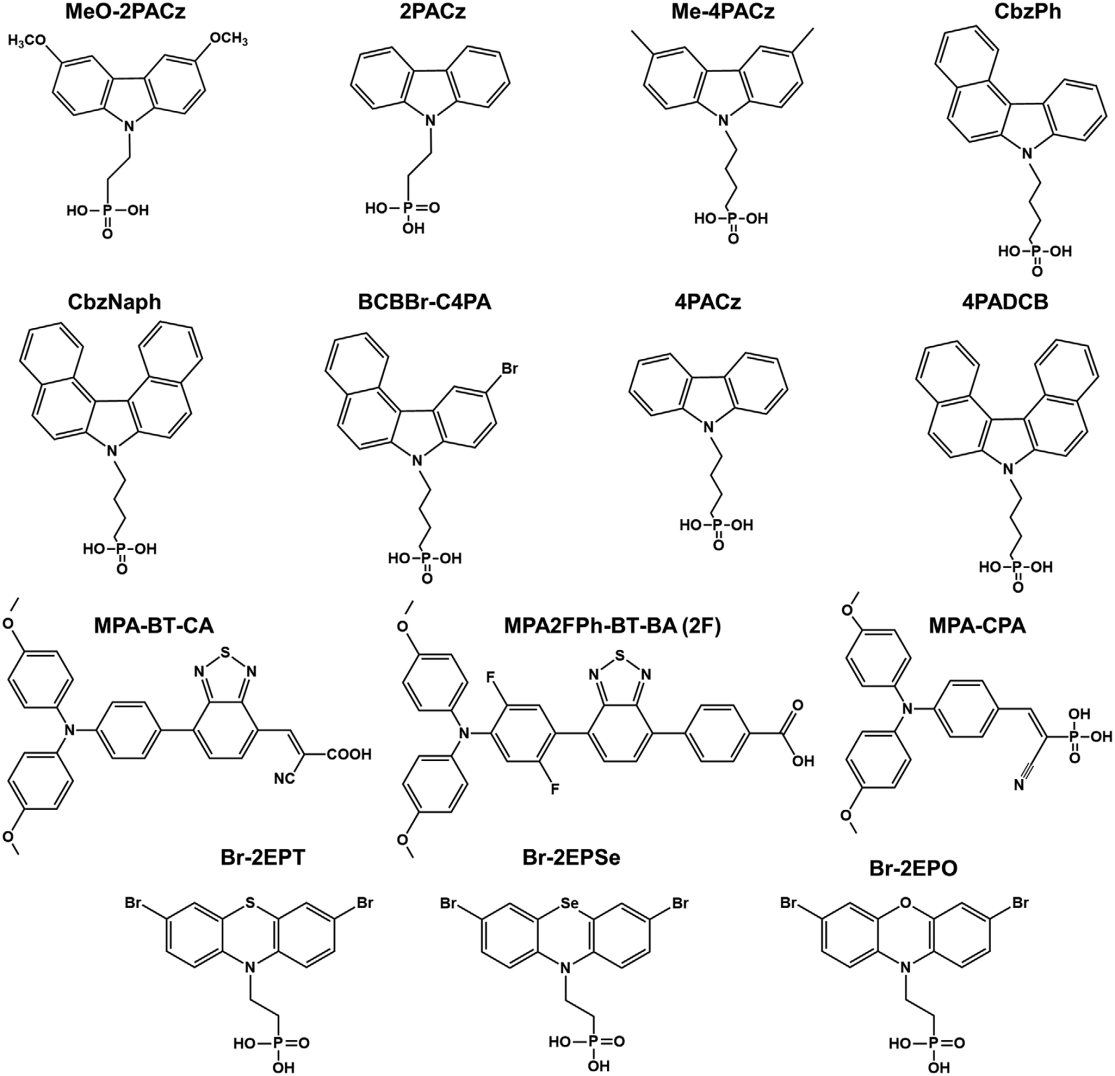
Molecular structures of green solvent‐processable SAMs HTL materials in *p*‐*i*‐*n* pero‐SCs.

Typically, SAMs consist of three parts: i) an anchoring group that chemically bonds the molecules to the substrate, ii) a connected spacer that determines the packing geometry of the SAMs, and iii) a functional group that modifies the surface and interface properties (**Figure** **9**).^[^ ^75^ ^]^ These SAMs can chemically bond to the surface of the ITO substrate to form an extremely thin and stable layer.^[^ ^76^ ^]^ The anchoring groups are usually polar carboxylic or phosphonic acid. According to HSP theory, the introduction of polar groups increases the molecular polarity, which provides good solubility for the SAMs in green solvents (e.g., IPA, EtOH). In addition, the functional groups are usually carbazole, MPA, phenothiazine, and their derivates. The photoelectric properties of these SAMs, such as conductivity and work function (W_F_), can be easily tuned by optimizing the functional groups. In summary, SAMs show several advantages in terms of substrate compatibility, simple dopant‐free processing protocols, and green solvent processability in *p*‐*i*‐*n* pero‐SCs.^[^ ^77^, ^78^, ^79^ ^]^ **Figure** **10** and **Table** **4** summarize the molecular structures of reported SAMs based on different functional groups for *p‐i‐n* pero‐SCs and detailed photovoltaic parameters.

**Table 4:** Detailed photovoltaic parameters of green solvent processable SAMs HTL materials in *p*‐*i*‐*n* pero‐SCs.

HTL material	Solvent	*V*_oc_ [V]	*J*_sc_ [mA cm^−2^]	FF [%]	PCE [%]	Ref.
2PACz	EtOH	1.188	21.9	80.2	20.9	[83]
2PACz:MeO‐2PACz	IPA	2.00	15.8	78.3	24.7	[84]
Me‐4PACz	TL	1.90	19.26	79.52	29.15	[79]
CbzNaph	IPA	1.17	24.69	83.39	24.1	[88]
BCBBr‐C4PA	EtOH	1.286	17.54	82.61	18.63	[89]
		1.264/ 0.855	16.42/ 13.50	83.17/ 77.84	26.24	
4PADCB	EtOH	2.11	15.37	83.13	27.01	[87]
MPA‐BT‐CA	EtOH	1.11	22.40	82.4	20.52	[94]
2F	TL	1.31	17.93	82.31	19.33	[95]
		0.872	32.55	81.89	23.24	
		2.130	15.52	82.36	27.22	
MPA‐CPA	EtOH	1.21	24.8	84.7	25.4	[45]
Br‐2EPT	EtOH	1.09	25.11	82	22.44	[90]
Br‐2EPSe	EtOH	1.12	24.49	82.86	22.73	[96]

#### 4.2.1 Carbazole‐Based SAMs

**Figure 11:**
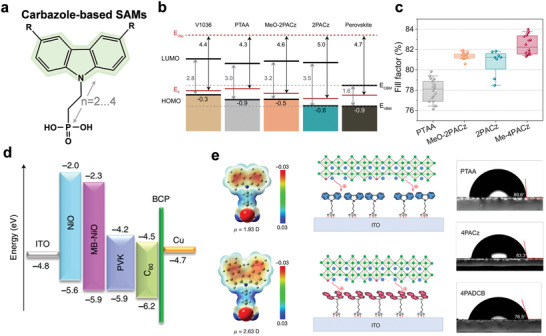
a) Chemical structure of a general carbazole‐based SAM, where ‐R representing ‐H, ‐OCH_3_, ‐CH_3_, etc. The numbers 2 or 4 denote the number of C atoms between the phosphonic acid anchor group and the conjugated carbazole main fragment. b) Schematic representation of the band edge positions of the investigated SAMs based on values from ultra‐violet photoelectron spectroscopy (UPS) measurements, referenced to the vacuum level. Reproduced with permission.^[^ ^83^ ^]^ Copyright 2019, Royal Society of Chemistry. c) Comparison of FF values of *p*‐*i*‐*n* pero‐SCs with different SAMs. Reproduced with permission.^[^ ^79^ ^]^ Copyright 2023, American Association for the Advancement of Science. d) Energy‐level diagram of the WBG pero‐SCs with NiO and MB‐NiO. Reproduced with permission.^[^ ^84^ ^]^ Copyright 2022, Springer Nature. e) Calculated ESP and contact angles of 4PACz and 4PADCB. Reproduced with permission.^[^ ^87^ ^]^ Copyright 2023, Springer Nature.

Carbazole is a common unit of traditional SAMs due to its hole‐selective properties,^[^ ^80^ ^]^ which was initially utilized in polymer light‐emitting devices (PLEDs)^[^ ^81^ ^]^ before being introduced in pero‐SCs (**Figure** **11**a).^[^ ^82^ ^]^ Albrecht and colleagues first synthesized two new simple SAMs based on carbazole cores and phosphonic acid anchoring groups, named MeO‐2PACz ([2‐(3,6‐dimethoxy‐9H‐carbazol‐9‐yl)ethyl]phosphonic acid) and 2PACz ([2‐(9H‐carbazol‐9‐yl)ethyl]phosphonic acid).^[^ ^83^ ^]^ As shown in Figure 11b, both MeO‐2PACz and 2PACz show *p*‐type characteristics as observed from the band edge positions, which indicates these SAMs are more hole‐selective than PTAA. Accordingly, these molecules can minimize the interfacial non‐radiative recombination losses effectively.^[^ ^83^ ^]^ In addition, thanks to the increased open‐circuit voltage (*V*_oc_), both MeO‐2PACz‐ and 2PACz‐based pero‐SCs surpass the efficiency of PTAA‐based devices, with the highest efficiency reaching 20.9% and a certified efficiency of 20.44%. More recently, they noted that simultaneously lowering the ideality factor and minimizing nonradiative interface recombination is crucial to obtaining a high *V*_oc_. Then, a new SAM with methyl group substitution was designed, Me‐4PACz ([4‐(3,6‐dimethyl‐9H‐carbazol‐9‐yl)butyl]phosphonic acid), which exhibited a rapid hole extraction ability as well as a lower ideality factor.^[^ ^79^ ^]^ These advances enabled the *V*_oc_ and fill factor (FF) to reach 1.23 V and 84% in *p*‐*i*‐*n* single‐junction pero‐SCs, respectively (Figure 11c). The obtained perovskite/silicon tandem solar cells (TSCs) delivered a certified PCE of 29.15%, which retained 95% of their initial efficiencies after 300 hours of operation.

Later, Tan et al. used a mixture of two hole‐selective molecules, 2PACz and MeO‐2PACz, to form a self‐assembled monolayer, which can bridge perovskite with a low‐temperature‐processed NiO nanocrystal film.^[^ ^84^ ^]^ Thanks to the improved energy‐level alignment (Figure 11d), reduced interfacial recombination, and enhanced hole extraction by the molecule‐bridged interface, the flexible wide‐bandgap (WBG) pero‐SCs demonstrated a substantial increase in PCE from 13.5% to 16.2%. Moreover, the resulting small‐area flexible all‐perovskite TSCs delivered a record PCE of 24.7% (certified 24.4%). Until now, *p*‐*i*‐*n* single‐junction pero‐SCs based on 2PACz and MeO‐2PACz, have been the most efficient devices, with PCEs exceeding 24% and 25%, respectively.^[^ ^85^, ^86^ ^]^

Although these carbazole‐based SAMs are promising hole‐selective materials for *p*‐*i*‐*n* pero‐SCs, their small dipoles cannot effectively modulate the W_F_ of the ITO substrate, which leads to high interfacial energy loss. To solve this problem, Jen et al. designed a novel carbazole‐derived SAM CbzNaph using helical *π*‐expansion.^[^ ^88^ ^]^ CbzNaph has a large molecular dipole (2.41 D) and aligns well with the highest occupied molecular orbital (HOMO) energy level (5.39 eV) of perovskite absorber, which can increase the W_F_ of ITO and reduce the interfacial energy loss. In addition, the strong π‐π interaction ensures more ordered and denser SAM. As a result, the champion *p*‐*i*‐*n* pero‐SCs achieved a PCE of 24.1% with improved device stability. By combining an asymmetric conjugation backbone and bromination strategy in the molecular design, Tang et al. reported a universal SAM‐based HTL material 4‐(10‐bromo‐7Hbenzo[c]carbazol‐7‐yl)butyl)phosphonic acid (BCBBr‐C4PA), which showed improved solubility and large dipole moment simultaneously.^[^ ^89^ ^]^ Furthermore, the lower‐lying HOMO energy level of BCBBr‐C4PA HTL reduces the energy offset with the perovskite valance band edge, which can realize greatly enhanced interfacial charge transfer and suppressed non‐radiative recombination losses. The resulting WBG pero‐SCs produced an impressive PCE of 18.63% with significantly enhanced operational stability, representing one of the highest PCEs for WBG pero‐SCs. A 0.5‐cm^2^ device could also maintain a high PCE (16.33%), which is a promising result for building larger devices. Crucially, these advantages were effectively translated into all‐perovskite TSCs, which generated an impressive PCE of 26.24%.

Unfortunately, achieving full coverage with certain SAMs, such as 2PACz and Me‐4PACz, on ITO can be difficult. These SAMs often have relatively poor solubility in alcohol due to the planar conjugated terminal groups, which can cause excessive molecular aggregation. As a result, these SAMs tend to precipitate rapidly during the solution evaporation process, which leads to non‐uniform coating of the SAM layers.^[^ ^90^, ^91^ ^]^ Moreover, the deficient surface wettability of these SAMs is a problem for the deposition of uniform and large‐scale perovskite films. Therefore, a rational molecular design is urgently needed to enable the large‐scale production of pero‐SCs. For example, Zhao et al. developed a SAM (4‐(7H‐dibenzo[c,g]carbazol‐7‐yl)butyl) phosphonic acid (4PADCB) as a hole‐selective layer. Here, two benzene rings were introduced into the carbazole group of commercial 4PACz to construct a new terminal group 7H‐dibenzo carbazole (DCB).^[^ ^87^ ^]^ The calculated electrostatic surface potential (ESP) results show that 4PADCB has a higher electron density on DCB, especially on the benzene rings near the interface (Figure 11e). This may enhance the interactions with the upper perovskite to facilitate interfacial charge transport. Furthermore, the constructed DCB moiety has a non‐coplanar screw‐shaped configuration because of the steric repulsive interaction between the terminal aromatic rings, which can hinder molecular aggregation. These advantages enable 4PADCB to show a more homogeneous anchoring on ITO than 4PACz, thus simultaneously improving the film coverage and surface wettability.^[^ ^92^ ^]^ As a result, 4PADCB (76.5°) shows a significantly reduced contact angle, compared with PTAA (89.6°) and 4PACz (83.3°). These advantages facilitate the subsequent growth of high‐quality WBG perovskite over a large area, and the suppressed interfacial non‐radiative recombination enables efficient hole extraction. As a result, a high efficiency of 27.0% (certified 26.4%) for centimeter‐scale all‐pero TSCs was reported.

#### 4.2.2 MPA‐Based SAMs

**Figure 12:**
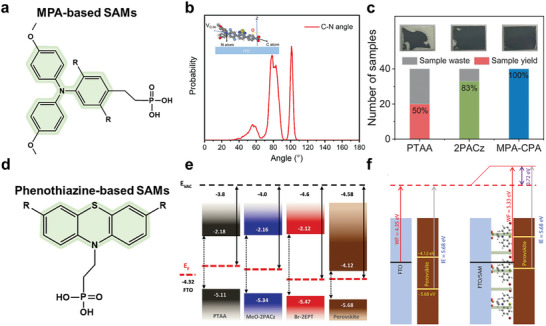
a) Chemical structure of a general MPA‐based SAM, where ‐R represents ‐H, ‐F. b) Molecular dynamics simulation of 2F on ITO substrate. Schematic and probability distribution of the C‐N axis for 2F. Reproduced with permission.^[^ ^95^ ^]^ Copyright 2023, Springer Nature. c) Fabrication yields of perovskite films on different HTLs without prewetting treatment and contact angles. Reproduced with permission.^[^ ^45^ ^]^ Copyright 2023, American Association for the Advancement of Science. d) Chemical structure of a general phenothiazine‐based SAM, with ‐R represents ‐H, ‐Br. e) Energetic alignment of different SAMs. Reproduced with permission.^[^ ^90^ ^]^ Copyright 2022, Wiley‐VCH. f) Schematic illustration of the energy level diagram of the FTO/perovskite interface before and after Br‐2EPSe modification. Reproduced with permission.^[^ ^96^ ^]^ Copyright 2022, Wiley‐VCH.

MPA is an organic amine with three phenyl groups surrounding the sp^2^ hybridized‐nitrogen atom. Due to the excellent electron‐donating and charge‐transport properties of MPAs, the majority of outstanding small molecular HTL materials are structured around a specific molecular scaffold, with varying numbers of MPA terminal groups (**Figure** **12**a).^[^ ^93^ ^]^ Guo et al. developed a new HTL material, MPA‐BT‐CA, comprising MPA as the D unit, BT as the A unit, and 2‐cyanoacrylic acid (CA) as the anchoring group. This material features alcohol processability, tunable energy levels, and defect passivation for the perovskite layer.^[^ ^94^ ^]^ As a result, the device, which is based on EtOH‐processed MPA‐BT‐CA HTL has a remarkably high PCE of 20.52% and good long‐term stability in ambient conditions. This is the first study that grafted the anchoring group from organic dyes to design efficient dopant‐free HTL materials that are low‐cost and feature environmentally friendly processability.

Inspired by this breakthrough, Zhao et al. designed a D‐A‐type molecule MPA2FPh‐BT‐BA (denoted as 2F), which served as an efficient hole‐selective contact that is compatible with both WBG and low‐bandgap (LBG) subcells for high‐performance all‐perovskite TSCs.^[^ ^95^ ^]^ By replacing the anchoring group 2‐cyanoacrylic acid in MPA‐BT‐CA with benzoic acid, 2F was obtained, and its molecular stacking behaviors could be fine tuned. Compared with MPA‐BT‐CA, 2F exhibits a narrower orientation distribution with the molecular backbone more parallel to the ITO surface, which is beneficial to the hole extraction (Figure 12b).^[^ ^97^, ^98^ ^]^ Furthermore, the introduction of F atoms results in a deeper highest HOMO level for 2F while also reducing the parasite absorption and enhancing defect passivation. Thanks to the strong interaction between 2F and Sn^2+^ in perovskites, 2F can regulate crystal growth and improve the film quality of the Sn‐containing LBG perovskite. Therefore, 2F‐treated WBG and LBG devices yielded efficiencies of 19.33% (certified 19.09%) and 23.24%, respectively, which made it possible to obtain all‐perovskite TSCs with an efficiency of 27.22% (certified 26.3%) and improved operational stability.

Amphiphilic small molecules, such as DMF and DMSO, are commonly used solvents for solution‐processed perovskites.^[^ ^99^ ^]^ However, most SAMs are either too hydrophobic to wet the perovskite precursor solution or chemically unstable when in contact with the perovskite.^[^ ^30^, ^100^ ^]^ Both factors can generate morphological, compositional, or electronic defects at the buried interface, which limit both photovoltaic efficiency and stability. To improve the performance of pero‐SCs, ideal SAMs should facilitate the growth of high‐quality perovskites with few nanovoids and deep‐level defects at the buried interfaces.^[^ ^101^, ^102^ ^]^ Considering the amphiphilic nature of perovskite precursor solution and the principle of “like‐dissolves‐like,” Wu et al. reported an amphiphilic molecular (2‐(4‐(bis(4‐methoxyphenyl)amino)phenyl)−1‐cyanovinyl)phosphonic acid (MPA‐CPA),^[^ ^45^ ^]^ which can enhance the perovskite deposition by providing a super wetting underlayer (Figure 12c). Furthermore, the CPA group exhibited improved hydrophilicity and defect passivation thanks to the synergistic coordination of the cyano and phosphonic groups with lead ions. In contrast to PTAA and 2PACZ, MPA‐CPA exhibited minimal contact angle (∼ 5°). This characteristic greatly facilitated the deposition of highly uniform perovskite films, especially on large‐area substrates. Without any modification layer on the HTL, the resulting *p*‐*i*‐*n* pero‐SCs achieved a certified PCE of 25.4% (0.08 cm^2^) with simultaneous improvement of both *V*_oc_ and FF. The encapsulated devices also exhibited high stability under both operational and damp heat testing conditions.

#### 4.2.3 Phenothiazine‐based SAMs

Today, most of the SAMs utilize electron‐rich heterocycles, such as carbazole or triphenyl amine‐based derivatives, as the core building blocks, which increases the synthesis cost of these molecules. As an alternative, phenothiazine has been extensively used in organic transistors,^[^ ^103^ ^]^ photovoltaics,^[^ ^104^ ^]^ and light‐emitting diodes.^[^ ^105^ ^]^ It represents a much cheaper building block that can be easily functionalized on multiple positions, with high chemical stability and remarkable hole mobility (Figure 12d).^[^ ^106^ ^]^ For example, Hong et al. utilized phenothiazine as the core unit and designed a novel halogenated phenothiazine‐based SAM, (2‐(3,7‐dibromo‐10H‐phenothiazin‐10‐yl)ethyl)phosphonic acid (Br‐2EPT).^[^ ^90^ ^]^ The Br‐2EPT SAM, a phenothiazine‐based compound featuring electron‐withdrawing bromine groups, exhibited superior alignment with perovskite absorbers, resulting in minimal nonradiative interfacial recombination loss compared to previously reported MeO‐2PACz‐based analogue and PTAA (Figure 12e). This dramatically improved charge extraction/transport as well as the device performance. The resulting pero‐SCs exhibited a PCE of up to 22.44% (certified 21.81%), with an average FF approaching 81%. The new SAM‐based device also demonstrated outstanding operational stability. In a word, the strong electron‐withdrawing Br atom and the more electronegative S atom increase the dipole moment of Br‐2EPT. It can effectively regulate the interface energetics and nonradiative loss pathways, thus determining the PCE and stability of pero‐SCs. In addition, these properties were also greatly affected by the functional groups of the SAMs that directly contacted the perovskite absorber. In light of the effect, Hong et al. further synthesized two variants of Br‐2EPT, 2‐(3,7‐dibromo‐10H‐phenoxazine‐10‐yl)ethyl)phosphonin acid (Br‐2EPO) and 2‐(3,7‐dibromo‐10H‐phenoselenazine‐10‐yl) ethyl)phosphonin acid (Br‐2EPSe), by incorporating O or Se, respectively, in place of S in the tricyclic aromatic functional group.^[^ ^96^ ^]^ All SAMs formed an energetically well‐aligned interface with the perovskite absorber (Figure 12f). Moreover, DFT calculations revealed the interaction energy between the Se‐containing SAM and perovskite absorber was the strongest in the series, which reduced the interfacial defect density, which, in turn, led to an extended charge carrier lifetime. As a result, the maximum PCEs of the pero‐SCs based on SAMs were 22.73%, 21.63%, and 21.02% for Br‐2EPSe, Br‐2EPT, and Br‐2EPO, respectively. The champion pero‐SCs that included Br‐2EPSe could retain ≈96% of their initial efficiencies after an MPP tracking test of 500 h without encapsulation under ambient conditions.

According to the above works, we summarized and proposed the SAMs molecular design guideline based on the HSPs principle to guide the green solvent‐processable dopant‐free organic HTL materials. Creating a suitable anchoring group that provides sufficient and homogeneous adsorption sites for robust and rapid adsorption is crucial to ensure the high uniformity of these monolayers. In addition to commonly used highly polar carboxylic or phosphoric acids as anchoring groups, it is necessary to explore novel anchoring groups that are currently unavailable, such as boric acid and anchoring groups with multiple anchoring sites, including multiple phosphonic acid anchoring groups.^[^ ^107^ ^]^ Moreover, the introduction of other functional groups, such as methoxy, cyanide, etc., can also fine‐tune the dipole moment. Finally, the connected spacer in the SAMs should also be carefully designed because it can optimize the molecular orientation and film geometry, which dramatically determine the optical and electrical properties of the SAM layers.

## 5 Summary and Perspectives

Before pero‐SCs can be successfully commercialized, it is necessary to transition from halogenated solvents to environmentally friendly green solvents in processing HTL materials. We reviewed the development of green solvent‐processable dopant‐free HTL materials concerning solvent selection and design. Because ideal green solvents generally have higher polarities than common toxic solvents, the increased polarity of the target molecule helps increase the solubility in these green solvents, according to HSP theory. More specifically, organic molecules with extended side chains, polar side chains, and asymmetric structures were carefully selected for green solvent processing. Particularly, asymmetric structure was considered a novel approach for increasing the solubility of HTL materials without sacrificing the hole mobility and stability.

Furthermore, D‐A‐type small molecule‐HTLs and SAM HTLs are regarded as excellent alternatives to polymer‐HTLs because of their well‐defined structure, easy purification, and well‐adjusted structure in *p*‐*i*‐*n* pero‐SCs. SAMs, in particular, present several advantages, including low material cost, compatibility with substrates, tunable bandgap, increased transmittance, and the ability to be processed with green solvents. These attributes have significantly propelled the rapid development of *p*‐*i*‐*n* pero‐SCs. Despite the rapid recent progress discussed in this review, some challenges and issues regarding green solvent‐processable dopant‐free HTL materials remain. Specifically, the following research directions are worthy of attention:

(1) As shown in the 3D HSP plot, ideal green solvents are associated with a higher *δ*_P_ than the common toxic solvents, and future HTL materials should exhibit higher *δ*_P_ and *R*_0_. Several challenges need to be addressed: One is the inferior charge transport associated with the short‐conjugated length and extended insulating portion. The other problem is the stability challenges due to the entropic effect of small molecules and the hygroscopic properties of the polar chain. Therefore, future molecular design could focus on developing asymmetric organic molecules with optimized polar chains in the conjugated backbone, which may extend to the robust polymeric system. Moreover, this approach can solve the transport and stability problem associated with dopant‐free HTL materials while maintaining a high solubility in green solvents.
(2) Regarding the molecular design for the ideal SAMs in the future, anchor groups in SAMs should be adjusted for specific substrates to fine‐tune W_F_. In addition, the functional groups have the benefits of facilitating perovskite deposition, delivering high‐quality perovskite films with few defects. Moreover, the connected spacer in the SAMs should also be carefully designed because it can optimize the molecular orientation and film geometry, which dramatically influence the optical and electrical properties of the SAM layers. Furthermore, an in‐depth study of the photophysical interaction and anchoring and its effect on trap passivation at the interface remains to be revealed for SAM in high‐performance *p*‐*i*‐*n* pero‐SCs. Interfacial studies at the atomic level may be the preferred way to disclose the mechanism. Hence, the following points should be focused on: i) the development of novel SAM structures specifically designed for pero‐SCs and comprehensively understanding of the individual roles of anchoring, the spacer, and the functional groups; ii) dual‐functional SAMs may be developed with electron‐ and hole‐ transport capability, to reduce the fabrication cost and simplify the process; and iii) for future practical manufacturing, a series of techniques could be used to realize the large‐scale uniform fabrication of these monolayers, including dip coating, blade coating, slot‐die coating and others. The key points to ensure high uniformity of these monolayers are creating a suitable substrate that can provide sufficient and homogeneous adsorption sites and designing efficient SAM molecules that can adsorb fast and robustly. Possible inhomogeneities such as unabsorbed SAM molecules can be removed by post‐treatment, such as solvent washing.^[^ ^108^ ^]^ Recently, a new work reported that SAMs can be fabricated by vapor‐phase deposition,^[^ ^109^ ^]^ which is also promising for future large‐scale manufacturing.

The strategies in this review will offer a rational guideline for developing greener solvents for high‐performance and environmentally friendly HTL materials, which contribute to the development and commercialization of pero‐SCs.

## Conflict of Interest

The authors declare no conflict of interest.
